# Natural Products with Potent Antimycobacterial Activity (2000–2024): A Review

**DOI:** 10.3390/molecules30183708

**Published:** 2025-09-12

**Authors:** Biniam Paulos, Daniel Bisrat, Maramawit Yonathan Yeshak, Kaleab Asres

**Affiliations:** Department of Pharmaceutical Chemistry and Pharmacognosy, School of Pharmacy, College of Health Sciences, Addis Ababa University, Addis Ababa P.O. Box. 1176, Ethiopia; biniam.paulos@aau.edu.et (B.P.); daniel.bisrat@aau.edu.et (D.B.); mariamawit.yonathan@aau.edu.et (M.Y.Y.)

**Keywords:** tuberculosis, mechanisms of action, minimum inhibitory concentration, selectivity index, natural products

## Abstract

Tuberculosis (TB), an infection caused by *Mycobacterium tuberculosis*, affects nearly one-third of the world’s population. It is estimated that TB infects around ten million people worldwide, with no less than two million fatalities annually. It is one of the treatable infections due to improved diagnostic tools and therapeutic agents. However, the disease remains a threat to humankind due to the emergence of multidrug- and extensively drug-resistant strains of *M. tuberculosis*. This has driven many researchers to look for new antitubercular medications with better efficacy, safety, and affordability. As has always been the case, natural products have provided huge potential as a source of remedies for various infectious and non-infectious diseases. This review aims to report discoveries and updates of antitubercular natural products with minimum inhibitory concentration (MIC) values of less than or 10 µg/mL or 50 µM and selectivity indices of greater than 10. The review discusses 36 naturally occurring compounds from various classes, isolated from both terrestrial and aquatic organisms, including higher plants and microorganisms. Perusal of the literature reveals that most of these promising compounds are alkaloids, terpenoids, steroids, and peptides. Rufomycin I, a cyclic heptapeptide from *Streptomyces* sp., showed potent activity against drug-sensitive and isoniazid-resistant *M. tuberculosis* H37Rv (MIC < 0.004 µM), surpassing isoniazid (MIC = 0.23 µM), likely by inhibiting ClpC1 transcription. Hapalindole A also displayed strong activity (MIC < 0.6 µM). Current TB drugs have become less effective; therefore, natural products such as hapalindole A and rufomycin I, owing to their potent activity, selectivity, and novelty, are increasingly recognized as potential lead compounds against TB.

## 1. Introduction

Tuberculosis (TB), one of the leading causes of death, is caused by *M. tuberculosis* [1,2]. *M. tuberculosis* (e.g., H37Rv and its attenuated counterpart H37Ra) is a classic slow-growing pathogen, with its doubling time in the laboratory being approximately 24 h, varying with the environment and with the time of visible colony development [3,4,5]. *M. avium*, being part of the *M. avium* complex (MAC), is also slow-growing and causes opportunistic pulmonary and disseminated infections, mainly among immunocompromised individuals [6]. In contrast, *M. smegmatis* is non-pathogenic and rapidly growing, possessing a 3-day colony growth rate and serving as a model in biosafety level 1 laboratories for the study of mycobacterial physiology and genetics [7]. Remarkably, using invasion assays of HEp-2 epithelial cells, *M. smegmatis* was not invasive or capable of multiplication unless it had been transformed with an invasive locus from *M. avium*, where it possesses the ability to invade and grow intracellularly, highlighting its universality as a substitute for the dissection of pathogenic traits [6].

*M. tuberculosis* H37Ra replicates the drug sensitivity profile of its virulent counterpart to most of the first- and second-line antitubercular agents. This makes *M. tuberculosis* H37Ra an excellent, safer substitute for in vitro drug screening and pharmacodynamic assays [8,9]. *M. smegmatis* can be used as a safe strain in a high-throughput model for screening anti-TB compounds with comparable MIC values to *M. tuberculosis* H37Rv for most TB drugs [7]. Other rapidly growing non-tuberculous species such as *M. aurum* have been observed to maintain drug susceptibility profiles similar to that of *M. tuberculosis* and be used as surrogate models for intracellular drug screening [9].

Around 2 billion people are latently infected with tuberculosis, yet only 10% of them develop active disease during their lifetime [10]. TB is treatable due to improved diagnostic tools and therapeutic agents in the 20th century [11,12]. Nevertheless, the emergence of HIV infection and the spread of multidrug-resistant TB (MDR-TB) in the 1980s and 1990s brought challenges in treatment [11]. Since 2000, global TB incidence has reduced by 1.7% per year, and age-standardized mortality rates fell from approximately 1.8 million deaths in 1990 to nearly 1.2 million in 2021. Incidents fell 11% between 2015 and 2020, but nevertheless, 10 million new cases continued each year. The COVID-19 pandemic reversed TB death gains, which increased to levels of 2017, as the rate fell by only 9% in 2020, considerably short of the 35% target. Inequities persist between regions, with Europe and parts of Africa recording steeper declines, while South/Southeast Asia and most low- and middle-income countries continue to bear heavy burdens. In 2023, TB once more became the leading infectious cause of death with 10.8 million cases and 1.25 million deaths [2].

The current pharmacotherapy of TB is challenged by the emergence of drug-resistant bacterial strains, side effects, and long duration of treatment. Hence, there is a pressing need to develop alternative treatments with novel modes of action, good oral bioavailability, shorter treatment duration, efficacy against MDR- and XDR-TB, broad availability and affordability, reduced pill burden, low dosing frequency, and minimal drug–drug interactions [13]. This could be achieved through looking into natural products or synthetic molecules. Natural products provide a large pool of molecules that could have an antimycobacterial effect or scaffold in anti-TB drug discovery [14].

Natural products could be screened for their antitubercular activity based on phenotypic (drug to target) or the target to drug screening [15,16]. Most anti-TB medications advanced to clinics through whole-cell screening, followed by analysis of the mechanism of action and identification of potential targets [17]. But even for early screening, antibacterial new drug discovery will be efficient and effective if it begins with a target that has been identified [15]. In this review, we shall discuss natural products with potent antimycobacterial activity reported in the English language from 2000 to 2024 with MICs less than 10 µg/mL or 50 µM and a selectivity index greater than 10. Data were collected from PubMed, Google Scholar, and relevant Internet sources by using keywords such as tuberculosis, mechanisms of action, minimum inhibitory concentration, selectivity index, and natural products. Mechanism of action and structure–activity relationships of the compounds will be discussed wherever reported.

## 2. Targets of Mycobacteria for Antitubercular Drug Discovery

### 2.1. Cell Wall of M. tuberculosis

The cell wall of *M. tuberculosis* is extremely intricately designed and contains elements that are involved in bacterial pathogenicity, host cell communication, and treatment resistance [18]. So, several medications have been produced that target the pathogen’s cell wall. The mycolic acid production pathway is one of the potential targets in the *M. tuberculosis* cell wall [19]. The production of mycolic acid is carried out by two enzymes, enoyl acyl carrier protein reductase (InhA) and -ketoacyl synthase, both of which are members of the fatty acid synthase (FAS) family [20]. Lysis of the bacterial cell results from the inhibition of these enzymes, which inhibits the bacteria’s ability to synthesize cell walls [11].

Since InhA is only present in bacteria, it makes it a good target for antibacterial treatments [21,22]. It is one of the enzymes responsible for producing mycolic acid, a component of the cell wall of *M. tuberculosis* [19]. There are three known binding sites on this NADH-dependent enzyme. Site I of NAD contains the tyrosine and ribose residues [23], Site II contains hydrophobic pockets that allow the binding of alkyl groups [24], and Site III has not been thoroughly explored but is thought to provide hydrophilic binding in which the phosphate group of NAD binds [25]. Isoniazid (INH) and ethionamide (ETH), two currently marketed medications, target this enzyme. Isoniazid is a prodrug that must be activated by catalase–peroxidase to form an unstable free radical, which binds to the NADH of InhA and forms a covalent bond, thereby inactivating the enzyme [22] and ultimately leading to mycobacterial cell death. To prevent the activation of INH, where the bacteria would otherwise develop drug resistance, the bacteria undergo a KatG mutation. Therefore, research on compounds that will prevent InhA from activating through KatG as antimycobacterial drugs will be ideal [26].

**Figure 1:**
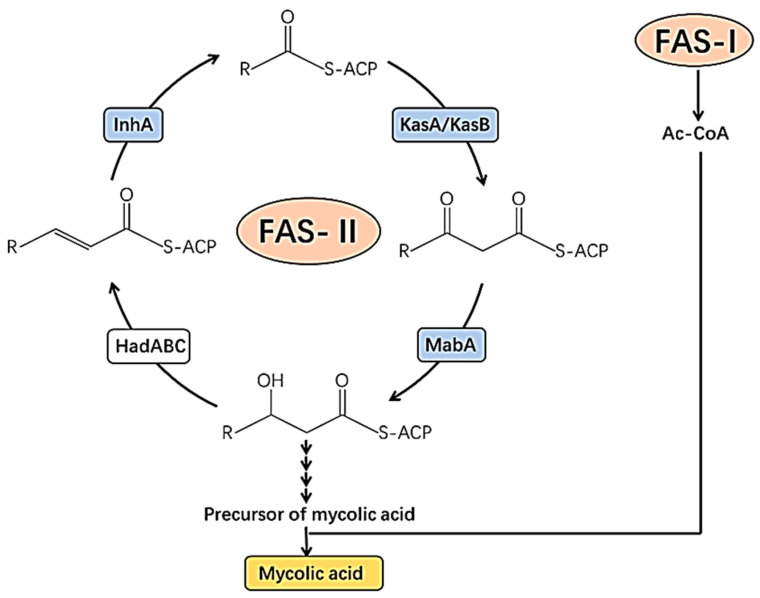
Potential target of the mycobacterial cell wall for drug targets. Ac-CoA, acetyl-coenzyme A; FAS-I, fatty acid synthase type I; HadABC, beta-hydroxyacyl-ACP dehydratase complex; InhA, 2-transenoyl-acyl carrier protein reductase; KasA/KasB, beta-ketoacyl-acyl carrier protein synthases A/B; MabA, beta-ketoacyl-acyl carrier protein reductase [28].

In addition to InhA, *β*-hydroxyacyl-acyl carrier protein (ACP) dehydratase complex (HadABC), *β*-ketoacyl ACP reductase (MabA), and -ketoacyl-acyl carrier protein (ACP) synthases (KasA and KasB, mtFabH, and mtFab) are enzymes involved in the production of mycolic acid (Figure 1) [27,28]. As a result, these enzymes could serve as a target for antimycobacterial drugs.

### 2.2. Nucleic Acids

Nucleic acids play an important role in the survival of mycobacteria. Notwithstanding this, there are few drugs approved for the treatment of TB on these targets [21].

#### 2.2.1. Purine and Pyrimidine Ribonucleotide Synthesis

In purine biosynthesis, an aminoimidazole moiety is produced and connected to a ribose by a sequence of reactions that are metabolized by the enzyme phosphoribosylpyrophosphate (PRPP) synthetase, which is dependent on inorganic phosphate, an essential metabolite for bacterial survival [22,23]. The C4 and C5 of imidazole then undergo cyclization, resulting in the production of inosine-5′-monophosphate (IMP), which is then transformed into guanosine 5′-monophosphate (GMP) by IMP dehydrogenase (IMPDH, GuaB) [29,30]. GuaB, one of the three IMPDH homologues that are currently known, is thought to be a viable therapeutic target because of its critical involvement in the metabolism of guanine nucleotides and as the cascade’s rate-limiting enzyme [21,25].

**Figure 2:**
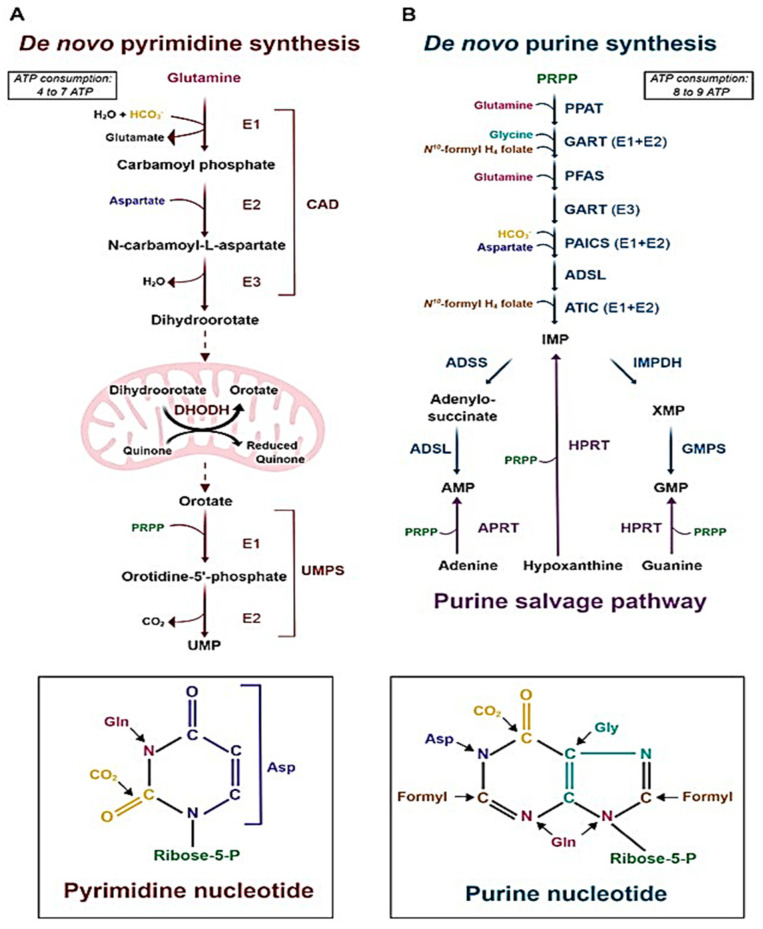
The de novo pyrimidine and purine biosynthesis pathways. (**A**) Diagram of the de novo pyrimidine biosynthesis pathway. Pyrimidine biosynthesis enzymes: CAD: Carbamoyl-Phosphate Synthetase 2, Aspartate Transcarbamylase, And Dihydroorotase; DHODH: Dihydroorotate Dehydrogenase; UMPS: Uridine Monophosphate Synthetase. (**B**) Diagram of the de novo and purine salvage pathways. Purine biosynthesis enzymes: PPAT: phosphoribosyl pyrophosphate amidotransferase; GART: Glycinamide Ribonucleotide Transformylase; PFAS: Phosphoribosylformylglycinamidine Synthase; PAICS: Phosphoribosylaminoimidazole Carboxylase and Phosphoribosylaminoimidazolesuccinocarboxamide Synthase; ADSL: Adenylosuccinate Lyase; ATIC: 5-Aminoimidazole-4-Carboxamide Ribonucleotide Formyltransferase; IMPDH: Inosine Monophosphate Dehydrogenase; GMPS: Guanine Monophosphate Synthase; ADSS: Adenylosuccinate Synthase; HPRT: hypoxanthine phosphoribosyltransferase; APRT: adenine phosphoribosyltransferase [30,33].

Uridine monophosphate, a precursor for pyrimidine nucleotides, is produced during the multi-enzymatic cascade reaction of pyrimidine biosynthesis [24]. Promising therapeutic targets have been investigated, including the cascade that orotate phosphoribosyltransferase (OPRT) catalyzes to transform orotate into orotidine 5′-monophosphate (OMP) [31,32]. Additionally, PRPP synthase, an enzyme that synthesizes PRPP, in turn, is necessary to produce both pyrimidine and purine nucleotides. Therefore, blocking this enzyme will also likely be a target for drugs (Figure 2) [29,30].

#### 2.2.2. Deoxyribonucleic Acid (DNA) Replication

**Figure 3:**
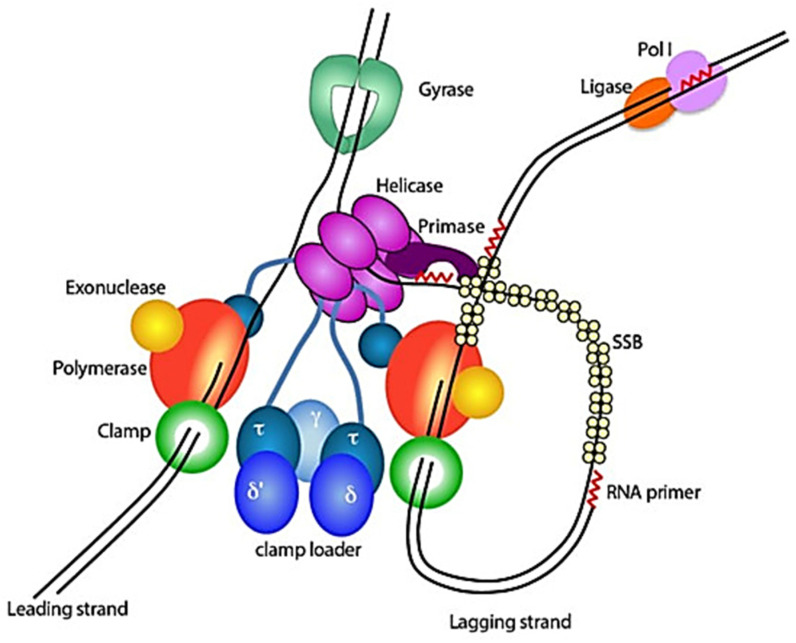
Schematic diagram of bacterial replisome: DNA gyrase unwinds supercoiling ahead of the fork, and DNA helicase unwinds DNA and invites primase to lay down RNA primers; leading and lagging strands are synthesized by DNA polymerases bound to clamps, which become loaded through a τ-containing clamp loader complex that bridges polymerases with helicase. SSB protects single-stranded DNA, and Polymerase I, and subsequently DNA ligase, removes primers and nicks to finish Okazaki fragments [37].

A multistep, multiprotein replisome in bacteria performs DNA replication in a highly controlled and coordinated manner [34]. These replisome proteins catalyze the production of RNA primers, clamp loading, DNA polymerization, and DNA unwinding [29]. Nearly 3950 genes make up the genome of *M. tuberculosis*, and 10% of them are necessary for the mycobacteria to survive [35,36]. The DnaA replication initiator, the primosomal (P) helicase, the DnaB helicase, the DnaG primase, the single-stranded DNA binding proteins (SSB), the clamp loader subunits, the DNA polymerases I and III, the DnaN-clamp, the DNA ligase I, and type I and II topoisomerases are among the 15 genes that are necessary for DNA replication (Figure 3) [34,37]. The single topoisomerase II class member, DNA gyrase (mostly DNA gyrA), is the only target for fluoroquinolones in the treatment of MDR-TB [38]. Therefore, there is a great potential to search for targets as DNA replication inhibitors and anti-mycobacterial drugs.

#### 2.2.3. DNA Repair

Due to the release of reactive oxygen species (ROS) and reactive nitrogen species (RNS), *M. tuberculosis* continuously sustains DNA damage in its host cell [39]. One of the RNS, nitrogen monoxide, interacts with oxygen to form nitrous anhydride, which then nitrosates amines and amides to produce potent DNA alkylating agents that endanger the survival of the bacteria because of genome instability [29]. Multi-enzymatic systems, including Nucleotide Excision Repair (NER) and Base Excision Repair (BER), recombination repair systems, and single proteins that directly reverse DNA damage, are all part of *M. tuberculosis* DNA repair mechanisms [40]. As a result, antimycobacterial agents that target this bacterial pathway will have potential. However, they possess a challenge of selectivity [29].

### 2.3. Protein Synthesis (RNA Translation)

The ribosome of *M. tuberculosis* is the key component in the translation of RNA into proteins [19]. The smaller subunit of ribosomes, known as 30S, contains 21 ribosomal proteins (RPs) and 16S ribosomal RNA (rRNA), which are used to decode messenger RNA (mRNA) sequences, and the larger subunit, known as 50S, contains 37 RPs and 20S and 5S rRNA and is used to form peptide bonds via the peptidyl transferase center (PTC) [19,41]. The 70S functional ribosome is made up of the two subunits [42]. The two subunits form the functional 70S ribosome [41]. Since these subunits’ interface is where the incoming transfer RNA (tRNA) passes via the aminoacyl (A-site), peptidyl (+P-site), and exit (E-site) sites, it is a crucial translational step [12]. The decoding center in the 30S and PTC and polypeptide exit site at the 50S subunit on the ribosome are prospective targets on the basis of the already available antibiotics such as aminoglycosides [43,44].

### 2.4. Energy Metabolism

**Figure 4:**
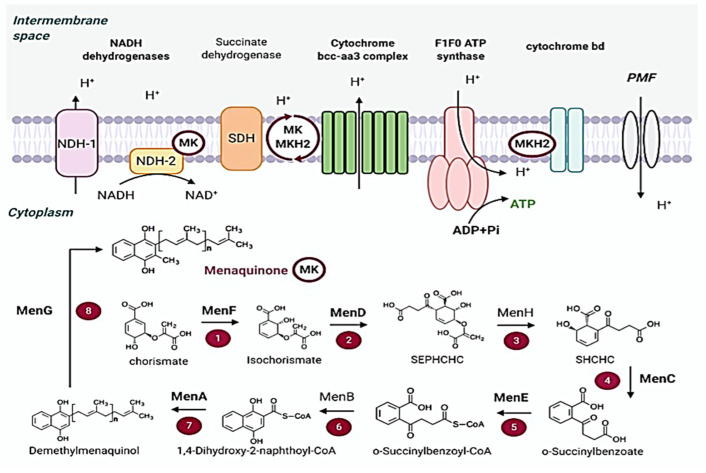
Schematic representation of ATP synthesis in *M. tuberculosis*: Menaquinone is reduced by NADH dehydrogenases (primarily NDH-2) and succinate dehydrogenases and donates electrons through terminal oxidases (Cyt-bcc-aa3 or Cyt-bd), generating a proton motive force that is used by F_1_F_0_-ATP synthase for the production of ATP [48].

*M. tuberculosis* produces ATP through oxidative phosphorylation, which is essential for growth and survival [45]. *M. tuberculosis* can produce ATP via substrate-level phosphorylation; however, it is insufficient [46]. Therefore, the primary source of energy for the bacteria is oxidative phosphorylation. NADH dehydrogenases and succinate dehydrogenases aid in the entry of the electron transport chain that transfers electrons to menaquinone, generating a protein complex and proton motive force (PMF), in the process of oxidative phosphorylation [46,47]. Adenosine triphosphatase (ATP synthase) uses the energy of this enzyme to produce ATP [35]. Since oxidative phosphorylation differs significantly between prokaryotes and eukaryotes, there are questions about the selectivity of antitubercular medications that target this route (Figure 4) [46,48]. Despite this, there are already available clinical and experimental medications that more specifically block the routes used by mycobacteria to synthesize ATP, revealing alternate targets for treating MDR and XDR infections.

## 3. Natural Products with Potent Antimycobacterial Activities

Numerous natural compounds derived from both aquatic and terrestrial sources, such as microbes and higher plants, have demonstrated potent anti-mycobacterial properties against various strains of mycobacteria. In vitro assays are used to determine the inhibitory concentration (IC) or minimum inhibitory concentration (MIC) of natural compounds [49,50]. For further study as potential anti-TB drug candidates, natural compounds with in vitro MIC ≤ 10 µg/mL or 50 µM are considered to have a significant antimycobacterial action. Therefore, these types of compounds are considered for further investigation, provided that their selectivity indices (SI) are greater than 10 [51,52]. This review focuses on natural products discovered between 2000 and 2024, with particular emphasis on compounds identified from 2020 to 2024 that exhibit potent in vitro activity against *M. avium*, *M. bovis*, *M. smegmatis*, and *M. tuberculosis* (Table 1). These products should demonstrate minimum inhibitory concentration (MIC) values of ≤10 µg/mL or 50 µM, along with a selectivity index (SI) greater than 10. The bioactive compounds meeting these criteria mainly fall within the classes of alkaloids, terpenes, peptides, and steroids.

### 3.1. Alkaloids

Alkaloids, which are a diverse group of nitrogen-containing natural products, have demonstrated promising potential against TB. Many plants, cyanobacteria, and marine-derived alkaloids have demonstrated potent antimycobacterial activity, with high selectivity indices (Table 1).

**Figure 5:**
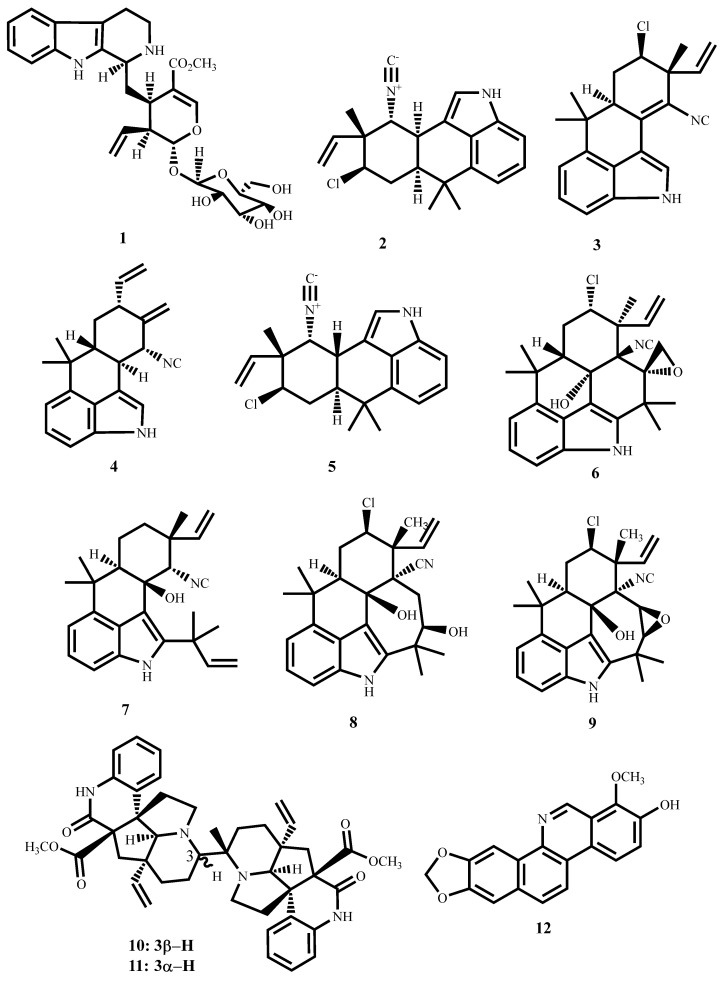
Structures of alkaloids (**1**–**12**) with potent antimycobacterial activity.

Strictosidine (**1**, Figure 5), a monoterpene indole obtained from the methanol extract of the leaves and twigs of *Psychotria nuda* (Cham. & Schltdl.) Wawra (Rubiaceae), showed an MIC value of 13.7 µM against *M. tuberculosis* H37Rv with a cytotoxic effect of 170.93 µM against RAW264.7 cells in the MTT assay [53].

The hapalindole-type alkaloids (**2**–**5**, Figure 5) and, fischambiguine B (**6**) and ambiguine isonitriles (**7**–**9**) from *Fischerella ambigua* and *Westiellopsis* species exhibit potent antimycobacterial activity (Table 1). Their antimycobacterial activity revealed key structure–activity relationships where epoxidation at C25–26, as in fischambiguine B (**6**), radically enhances potency and selectivity against *M. tuberculosis* H37Rv (MIC ~2 µM; SI > 60). Similarly, ambiguine E isonitrile (**9**) also contains an epoxy ring, which is fused with the seven-membered E ring. However, its activity dropped by 10-fold. This implies that the spiro-epoxy containing six six-membered rings is essential for activity. Moreover, chlorination at C13 is non-influential on activity. Hapalindole G, which lacks the epoxide but remains chlorinated, is moderately active and less selective (SI > 18). For ambiguine isonitriles, compounds C and M are moderately active against *M. tuberculosis* (MIC = 7 µM; SI ≈ 11), but ambiguine E is exceptionally selective for *M. smegmatis* (MIC = 1.4 µM; SI = 30.42), while being less active against *M. tuberculosis*. Taken together, these findings point to the significant role played by the epoxide moiety in optimizing antimycobacterial activity and reaffirm the isonitrile core as being requisite for lower activity [54,55,56,57].

Suadamin A (**10**) and Suadamin B (**11**) are dimeric monoterpenoid quinoline alkaloids, which were obtained from *Melodinus suaveolens* (Hance) Champ, ex Benth. Both compounds exhibited antimycobacterial activity against *M. tuberculosis* H37Rv, with Suadamin A having a significantly lower MIC (6.76 μM) (Table 1). The SAR analysis revealed that their stereochemistry at the C-3 position is the main cause of the difference in potency [58]. Stereochemistry often dictates drug–target interactions since substituent spatial arrangements determine how the compound fits into the binding site of its mycobacterial target. For Suadamins, the C-3 stereogenic center controls the orientation of the most critical functional groups in the dimeric framework that influence hydrogen bonding, hydrophobic contact, and steric complementarity with the bacterial enzyme or membrane constituent responsible for the antimycobacterial effect.

For Suadamin A, the C-3 stereochemistry facilitates the quinoline and monoterpenoid functionalities to assume a conformation that better complements the mycobacterial target’s binding pocket, increasing activity. Conversely, the other stereochemistry of Suadamin B likely causes steric clash or pharmacophore misalignment, consequently reducing binding affinity and antimycobacterial activity. This conforms to broad general medicinal chemistry principles where stereochemical differences, occasionally even a sole chiral center, can result in large variations in bioactivity, as in thalidomide enantiomers or quinine versus quinidine [74,75]. Thus, the higher selectivity index and potency of Suadamin A emphasize the importance of stereochemistry in natural product drug discovery. The findings suggest that precise stereochemical control at important centers such as C-3 should be considered in any possible synthetic or semi-synthetic modification of suadamins and related alkaloids for drug development against tuberculosis.

Another isoquinoline-based alkaloid, decarine (**12**), isolated from the roots of *Zanthoxylum capense* (Thunb.) Harv., has been shown to exhibit substantial activity against *M. tuberculosis* H37Ra and H37Rv, with MIC values of 9.71 μM and 5.01 μM, respectively (SI = 41.2) [59].

### 3.2. Simple Amide and Peptides

**Figure 6:**
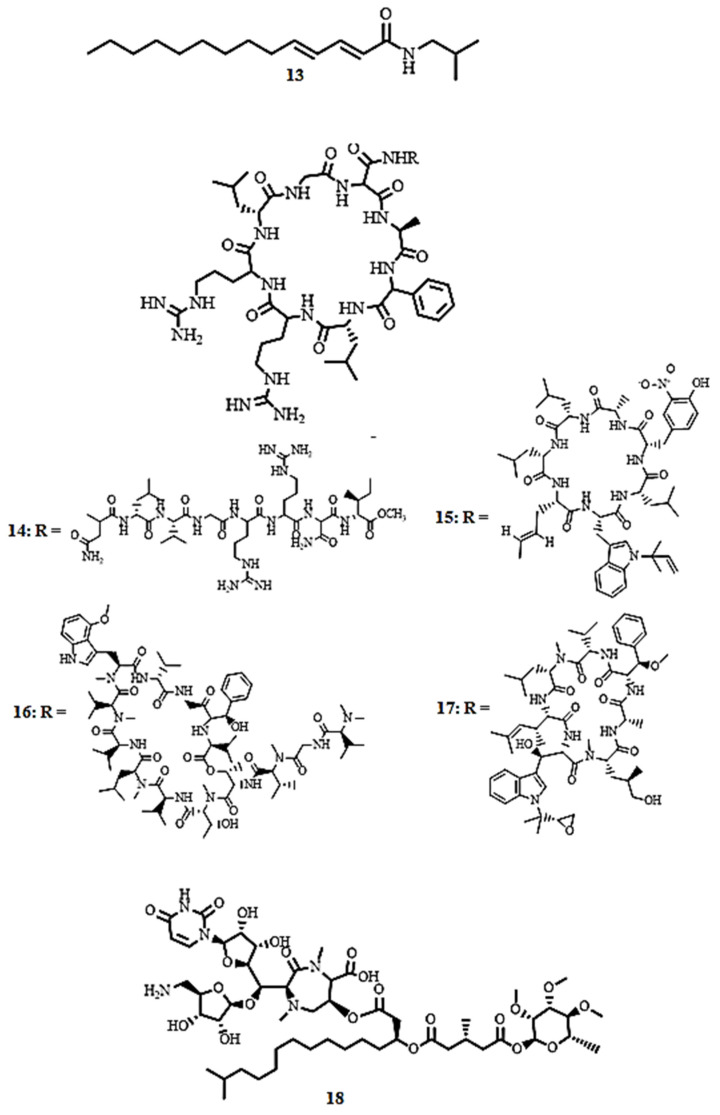
Structures of an amide (**13**) and peptides (**14**–**18**) with potent antimycobacterial activity.

Cyclic peptides and usual amides have become potential antimycobacterial drugs, showing potent activity against both drug-sensitive and drug-resistant *M. tuberculosis* strains. The cyclic peptides and amides contained in this review are mostly derived from *Streptomyces* and *Zanthoxylum capense* (Thunb.) Harv (Table 1). They have been shown to exhibit bactericidal activity at low MIC values with low cytotoxicity. The mechanisms of action of cyclic peptides and amides, particularly their inhibition of ClpC1 ATPase, provide a new direction for the discovery of therapeutics against tuberculosis.

(2*E*,4*E*)-N-Isobutyl-2,4-tetradecadienamide (**13**, Figure 6), a simple amide isolated from the roots of *Zanthoxylum capense* (Thunb.) Harv demonstrated antimycobacterial activity against *M. tuberculosis* H37Rv with MIC values of 5.73 µM (SI = 38.2), in the broth micro-dilution method [59].

Lassomycin (**14**, Figure 6), a cyclic peptide isolated from *Lentzea kentuckyensis*, exhibits potent bactericidal activity against *M. smegmatis*, *M. tuberculosis* H37Rv, and *M. avium* subsp. *paratuberculosis*, including drug-resistant (MDR) and extensively drug-resistant (XDR) strains, with MICs ranging from < 0.004 to 1.66 µM and low cytotoxicity against human NIH 3T3 and HepG2 cells (IC_50_ = 187.34 µM) [16]. Molecular docking studies reveal that lassomycin is sequestered in the acidic ATPase pocket of the ClpC1 caseinolytic protein, inhibiting its proteolytic activity [9]. Cyclic peptides rufomycin I (**15**, MIC < 0.004 µM, SI > 250), ecumicin (**16**, MIC = 0.16 µM, SI > 192), and cyclomarin A (**17**, MIC = 0.094 µM, SI = 26.7), derived from *Streptomyces* sp., were active against drug-sensitive and drug-resistant *M. tuberculosis* H37Rv, *M. smegmatis*, and *M. avium*, with rufomycin I being more potent than isoniazid (MIC = 0.23 µM) [60,61]. These cyclic peptides targeting ClpC1 represent a novel and promising antibacterial approach. Instead of classical enzyme inhibition, they disable the essential proteolytic machinery of *M. tuberculosis* by decoupling or deregulating critical functional cycles. This innovative approach, whether through uncoupling ATP hydrolysis from proteolysis, selectively inhibiting substrate turnover, or misdirecting the protease, leads to a lethal collapse of proteostasis [60,61]. Consequently, these compounds emerge as lead candidates for the development of new anti-TB drugs, offering a unique strategy to treat both drug-sensitive and drug-resistant mycobacterial infections.

Caprazamycin B (**18**, Figure 6), a novel peptide from one of the *Streptomyces* isolates, showed excellent inhibitory activity against *M. tuberculosis* H37Rv Kurono and *M. bovis* Ravenel, with the MIC value of 2.73 µM. The compound also showed a very good safety profile with no in vivo cytotoxicity observed in mice at 4368.4 µM, which corresponds to a selectivity index (SI) value above 1500 [62]. These results bring into focus caprazamycin B as a promising molecule for further development as a selective antitubercular agent.

### 3.3. Terpenoids

**Figure 7:**
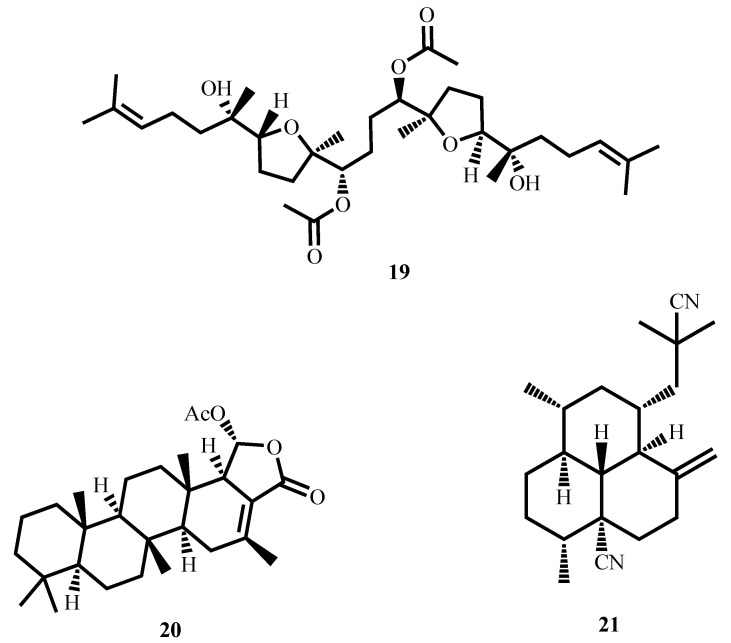
Structures of terpenoids (**19**–**21**) with potent antimycobacterial activity.

Strong activity against *M. tuberculosis* H37Rv and *M. tuberculosis* M299 was demonstrated by the squalene-type triterpenoid eurylene (**19**, Figure 7), isolated from the roots of *Homalolepis suffruticosa* (Engl.) Devecchi & Pirani, with MICs of 2.35 µM and 3.36 µM, respectively. These compounds had better selectivity (SI > 20) when tested with the MTT method [63].

12-Deacetoxyscalarin 19-acetate (**20**), a scalarane type sesterterpenoid, was isolated from marine sponges. It is an extremely potent antimycobacterial compound against *M. tuberculosis* H37Ra with an MIC of 4 µM (Table 1) [64]. Its action mode is the inhibition of farnesyltransferase, an enzyme catalyzing post-translational modification of proteins by attaching a farnesyl group [76]. This inhibition also prevents the functioning of membrane-associated proteins and leads to compromised bacterial membrane integrity and subsequent cell death. This positions 12-deacetoxyscalarin 19-acetate as a lead compound in the design of novel antimycobacterial agents targeting membrane-associated processes.

Nieves et al. [65] reported that (−)-8,15-diisocyano-11(20)-amphilectene (**21**) exhibited strong antimycobacterial activity against *M. tuberculosis* H37Ra (MIC = 9.8 µM) with moderate selectivity toward mammalian cells (SI = 10.2).

### 3.4. Steroids

Steroidal compounds have displayed potent inhibitory activity against *M. tuberculosis*, including the drug-sensitive strains, particularly the triterpene and phytosterol derivatives. The steroids, including saringosterol and stigmastene, were made from plant and marine algae, and had relatively low MIC values, some equaling the potency of standard drugs such as rifamycin. The selectivity indices and structure–activity relationships were promising, particularly in terms of the role of unsaturated ketone moieties, indicating the steroids could have utility as scaffolds for anti-TB drug development (Table 1).

**Figure 8:**
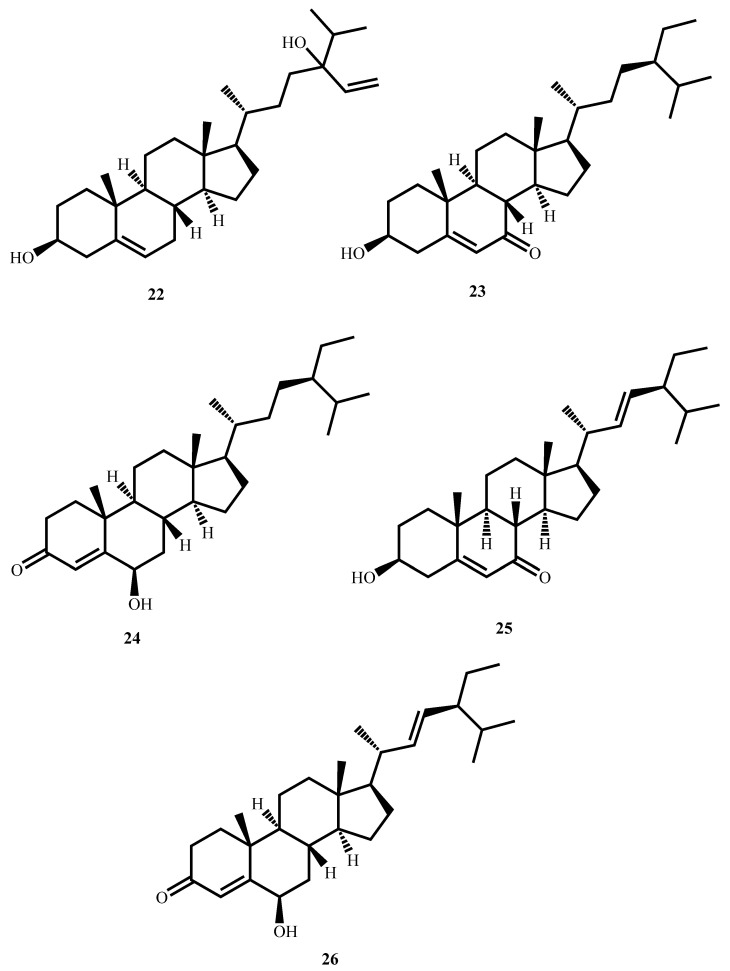
Structures of steroids (**22**–**26**) with potent antimycobacterial activity.

Saringosterol (**22**, Figure 8), a triterpene steroid from the brown alga *Lessonia nigrescens*, possesses potent antimycobacterial activity against *M. tuberculosis* H37Rv (MIC = 0.58 µM). The single 24*S* and 24*R* epimers of saringosterol were also analyzed in the study, and they presented MIC values of approximately 2.33 µM and 0.29 µM, respectively, indicating that the 24*R* epimer is more active. The findings indicate the promise of saringosterol and its epimers as potential leads for the development of new antimycobacterial agents [66].

Bioassay-guided fractionation of the aerial parts of *Thalia multiflora* Horkel ex Koernicke (Marantaceae) using dichloromethane–methanol (1:1) gave stigmast-5-ene-3β-ol-7-one (**23**, MIC = 4.62 µM), stigmast-4-ene-6β-ol-3-one (**24**, MIC = 10.07 µM), stigmast-5,22-diene-3β-ol-7-one (**25**, MIC = 2.34 µM), and stigmast-4,22-diene-6β-ol-3-one (**26**, MIC = 2.35 µM) against *M. tuberculosis* H37Rv [67]. Structure–activity relationship studies revealed that the presence of an α,β–unsaturated ketone on ring A or ring B significantly enhances antitubercular activity. Of interest, all of the compounds were found to be highly selective against the pathogen with selectivity indices greater than 10 against Vero cells, denoting low cytotoxicity against mammalian cells. The findings suggest that these stigmastane analogs are good prospects for further development as selective antitubercular drugs.

### 3.5. Miscellaneous Compounds

There exists a wide variety of structurally diverse natural products with antimycobacterial activities, including fatty acid derivatives, polyketides, lactones, organic acids, and others. Certain compounds (e.g., falcarindiol and micromolide) exhibited low values of MIC with selectivity indices and good mechanisms of action (e.g., inhibition of the biosynthesis of para-aminobenzoate (PABA) and succinate dehydrogenase). The potent activity of these compounds against drug-sensitive and resistant strains of *M. tuberculosis* supports and may indicate their potential for use in future explorations for anti-TB drugs (Table 1).

**Figure 9:**
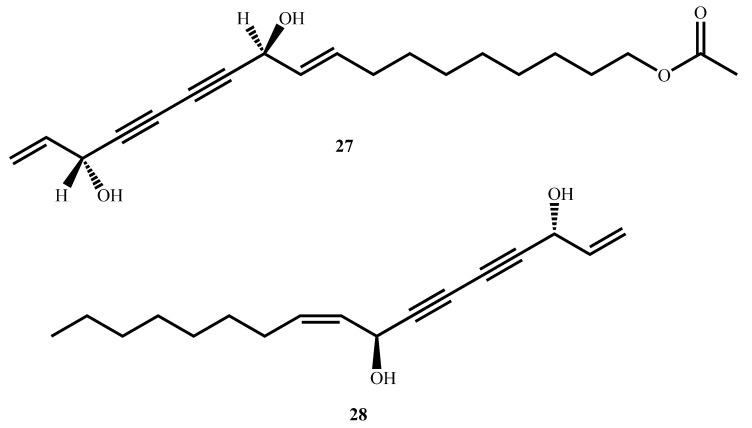
Structures of polyacetylenes (**27**, **28**) with potent antimycobacterial activity.

11(*S*),16(*R*)-Dihydroxy-octadeca-9Z,17-dien-12,14-diyn-1-yl acetate (**27**, Figure 9) and falcarindiol (**28**), isolated from the methanol extract of *Angelica sinensis* (Oliv.) Diels roots, were robustly antimycobacterially active against *M. tuberculosis* Erdman, with MICs of 4.21 µM and 23.04 µM, respectively [68]. Both compounds showed high selectivity against the pathogen, with selectivity indices of >117 and >19, respectively, indicating minimal toxicity toward mammalian cells. Structure–activity relationship (SAR) investigations revealed that antimycobacterial activity is maintained with the hydroxyl group at C16 and with an unsaturation at C9–C10 [68]. The findings suggest that the compounds are good candidates for further development as selective antimycobacterial drugs and provide valuable information for designing derivatives with improved efficacy and safety profiles [40].

**Figure 10:**
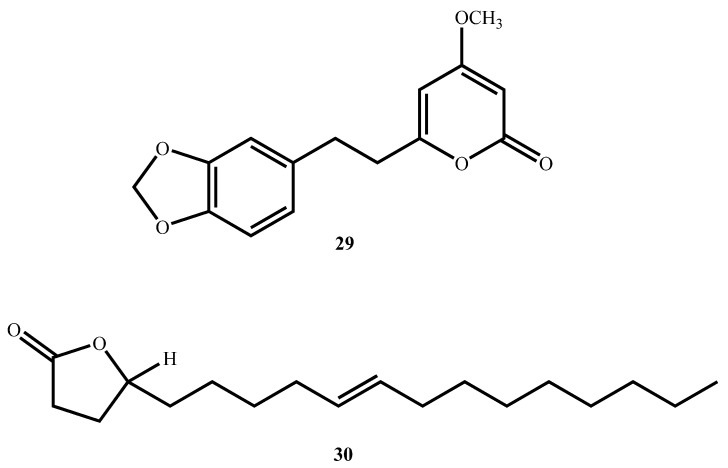
Structures of lactones (**29**, **30**) with potent antimycobacterial activity.

Two lactones with potential antimycobacterial activity were isolated. Kavalactone 5,6-dehydro-7,8-dihydromethysticin (**29**, Figure 10) from the leaves and stems of *Piper sanctum* (Miq.) Schltdl. ex C. DC. inhibited *M. tuberculosis* H37Rv with an MIC of 12.04 µM [69]. Micromolide (**30**), a γ-lactone oleic acid derivative from *Micromelum hirsutum* Oliv., was even more active (MIC = 5.35 µM) with a high SI value (63) against Vero cells. Micromolide was further demonstrated to exhibit robust efficacy under more physiological conditions with an EC_90_ of 19.97 µM against the virulent Erdman strain in J774 mouse macrophages, and thus it presents as a promising lead for future in vivo studies [70].

**Figure 11:**
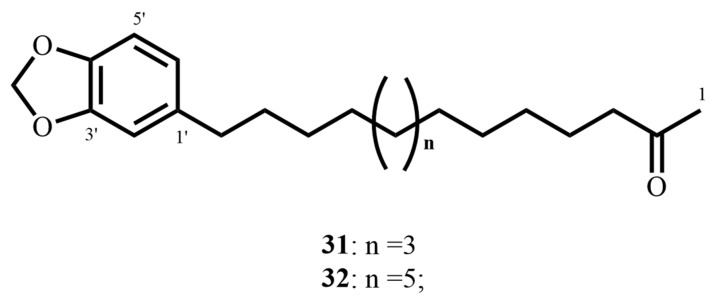
Structures of aryl-alkyl ketones (**31**, **32**) with potent antimycobacterial activity.

Besides these lactones, two aryl-alkyl ketones, 2-oxo-14-(3′,4′-methylenedioxyphenyl)-tetradecane (**31**, Figure 11) and 2-oxo-16-(3′,4′-methylenedioxyphenyl)-hexadecane (**32**), were identified in *P. sanctum*. Both of these compounds inhibited *M. tuberculosis* H37Rv with MIC ranges of 12.04–18.81 µM and excellent selectivity indices greater than 10 against Vero cells. This combination of potent antimycobacterial activity with minimal cytotoxicity positions the aryl-alkyl ketones, as well as the lactones, very favorably as scaffolds for selective antitubercular drug development [69].

**Figure 12:**
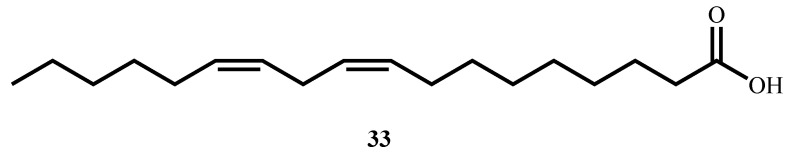
Structure of a fatty acid (**33**) with potent antimycobacterial activity.

Linoleic acid (**33**, Figure 12), isolated from the CH_2_Cl_2_ extract of the stem bark of *Warburgia ugandensis*, showed powerful activity against *M. aurum* and *M. phlei* with MIC values of 14.27 µM. In addition, it showed activity against *M. fortuitum* (MIC = 28.53 µM) [71]. *M. tuberculosis* has evolved to utilize long-chain fatty acids (LCFAs) as a preferred carbon source. However, LCFAs have long been known to exhibit bactericidal effects against *M. tuberculosis* in vitro by disrupting the bacterial membrane, leading to hyperpolarization and subsequent cell death. A deeper understanding of how *M. tuberculosis* regulates the uptake and metabolism of LCFAs, and how these pathways can be therapeutically targeted, is essential for the development of novel anti-TB agents.

**Figure 13:**
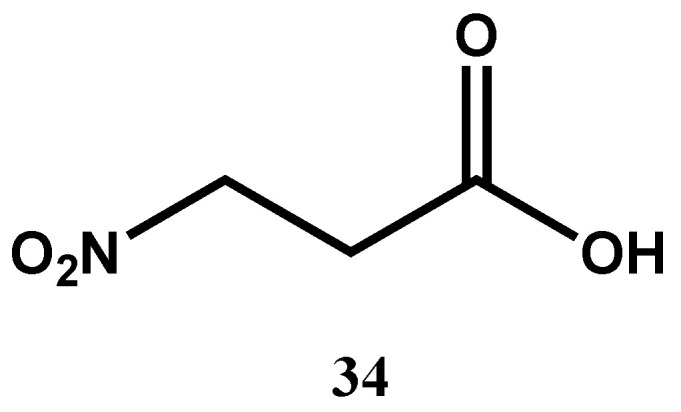
Structure of a nitroacid (**34**) with potent antimycobacterial activity.

3-Nitropropionic acid (**34**, Figure 13) isolated from the endophytic fungal strain *Phomopsis* sp. strain usia5 was found to be a potent antimycobacterial with an MIC of 3.3 μM against *M. tuberculosis* H37Ra, without any detectable cytotoxicity against Vero cells [72]. Its mode of action was predicted to inhibit succinate dehydrogenase, a critical enzyme of the tricarboxylic acid (TCA) cycle and the electron transport chain [77]. By inhibiting succinate dehydrogenase, 3-nitropropionic acid decouples both energy generation and redox homeostasis in mycobacteria, which are essential for their survival and persistence. Its strong antimycobacterial activity, low host toxicity, and distinct mechanism of action compared to conventional antitubercular drugs make it a promising lead compound for the development of novel therapeutics and validate energy metabolism as a target for antibacterial drug discovery.

**Figure 14:**
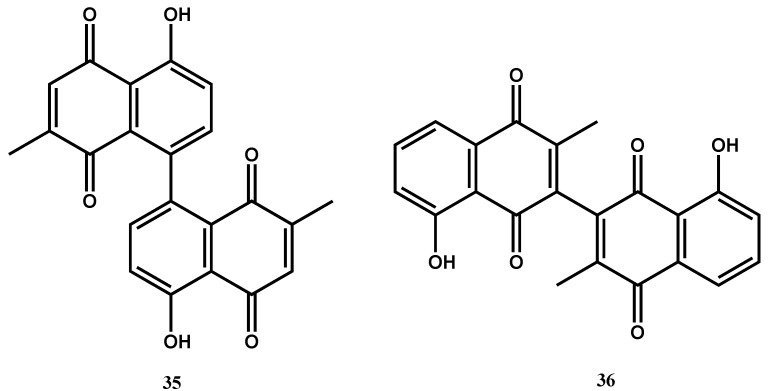
Structures of naphthoquinones (**35**, **36**) with potent antimycobacterial activity.

Maritinone (**35**, Figure 14) and 3,3′-biplumbagin (**36**), naphthoquinones isolated from the n-hexane extract of the stem bark of *Diospyros anisandra* S.F.Blake, demonstrated strong activity against drug-sensitive *M. tuberculosis* H37Rv with an MIC value of 8.34 µM each (SI > 10; against Vero cells) [73]. The above anthraquinones have been found to inhibit flavin-dependent thymidylate synthase ThyX, which is responsible for catalyzing the essential methylation of dUMP to dTMP for DNA synthesis in *M. tuberculosis* [78]. Targeting ThyX is an excellent strategy for antitubercular lead optimization: the enzyme is essential for mycobacterial replication and viability in latent and intracellular infections, yet absent in humans, thereby offering highly selective inhibition with low host toxicity. Structural and mechanistic differences between ThyX and the human thymidylate synthase (ThyA), including flavin cofactor dependency and distinct active-site topology, also allow for the potential development of selective inhibitors, providing a promising avenue toward novel tuberculosis treatments [79].

## 4. Conclusions

Existing therapies have had little impact on the management of TB on a worldwide scale, and new regimens are urgently required. The emergence of MDR and XDR forms of *M. tuberculosis* has rendered many of the existing therapeutic agents ineffective, yet again emphasizing the need for drugs with novel modes of action and targets. Natural products from a vast array of sources have been incredibly promising as antimycobacterial compounds with potential clinical use. Agents such as hapalindole A (MIC < 0.6 µM; SI > 53), lassomycin (MIC = 0.78–1.56 µg/mL; SI > 224), rufomycin I (MIC < 0.004 µM; SI > 250), ecumicin (MIC = 0.26 µg/mL; SI > 192), and cyclomarin A (MIC = 0.094 µM; SI = 26.7) have shown excellent in vitro activity with high selectivity indices, making them candidates of interest for drug discovery. These findings suggest that natural products remain one of the richest sources of leads for new drugs and will undoubtedly remain involved in antimycobacterial drug discovery. But optimization of such discoveries into drugs of clinical utility is highly constrained. Most anti-TB natural product leads are found through phenotypic screens versus rational target-based technologies, which can be inhibitive to hit-to-lead optimization. Other difficulties include natural product structural complexity, sustainable large-scale production challenges, inadequate solubility and biocompatibility, potential host toxicity, and yield variability depending on environmental or seasonal parameters, with the further barrier of prolonged preclinical screening mechanisms.

These difficulties can be addressed with multidisciplinary approaches involving natural product chemistry, microbiology, pharmacology, and computational sciences. Semi-synthetic derivatization of lead-like scaffolds can enhance pharmacokinetic properties, and metabolomics, genomics, synthetic biology, and artificial intelligence have the potential to accelerate lead discovery and optimization. Representative *M. tuberculosis*-specific screening platforms and in silico ADMET and molecular docking studies should be incorporated early in the drug development pipeline to enhance efficiency. In addition to this, sustainable sourcing schemes such as microbial fermentation, endophytic culture, and plant tissue culture need to be guaranteed to provide compound availability consistency. Finally, natural products provide unparalleled chemical diversity and new mechanisms of action, but their complete potential as sources for TB drug discovery depends on high-tech, cooperative, and technologically synergistic approaches. With sustained investment and global cooperation, the projects could finally offer safe, affordable, and cost-effective anti-TB drugs to counteract the increasing menace of drug-resistant TB.

## Figures and Tables

**Table 1:** Summary of natural products with potent antimycobacterial activity and selectivity indices greater than 10 from 2000 to 2024.

Compound’s Name (Class of Compounds)	Molecular Formula	Molecular Weight (g/mol)	Source	MIC in µM (Mycobacterial Strain)	Anti-Mycobacterial Assay Methods	Cytotoxicity in µM (Cell Line)	Selectivity Index	Ref
Strictosidine (**1**) (Alkaloid)	C_27_H_34_N_2_O_9_	530.6	*Psychotria nuda* (Cham. & Schltdl.) Wawra	13.7 (*M. tuberculosis* H37Rv)	MTT	170.93 (RAW264.7 cells)	12.47	[53]
Hapalindole A (**2**) (Alkaloid)	C_21_H_23_ClN_2_	338.9	*Westiellopsis* sp. and *Fischerella muscicola*	<0.6 (*M. tuberculosis* H37Rv)	MABA	31.9 (Vero cells)	>53	[54,55,56,57]
Hapalindole I (**3**) (Alkaloid)	C_21_H_21_ClN_2_	336.9		2 (*M. tuberculosis* H37Rv)		>100 (Vero cells)	>50	
Hapalindole X (**4**) (Alkaloid)	C_21_H_22_N_2_	302.42		2.5 (*M. tuberculosis* H37Rv)		35.2 (Vero cells)	14.08	
Hapalindole G (**5**) (Alkaloid)	C_21_H_23_ClN_2_	338.9		6.8 (*M. tuberculosis* H37Rv)		>128 (Vero cells)	>18.8	
Fischambiguine B (**6**) (Alkaloid)	C_26_H_29_ClN_2_O_2_	436.18		2 (*M. tuberculosis* H37Rv)		>128 (Vero cells)	>64	
Ambiguine C isonitrile (**7**) (Alkaloid)	C_26_H_32_N_2_O	388.5		7.0 (*M. tuberculosis* H37Rv)		78.3 (Vero cells)	11.25	
Ambiguine M isonitrile (**8**) (Alkaloid)	C_26_H_31_ClN_2_O_2_	439.0	*Fischerella ambigua*	7.5 (*M. tuberculosis* H37Rv)		79.8 (Vero cells)	10.64	
Ambiguine E isonitrile (**9**) (Alkaloid)	C_26_H_29_ClN_2_O_2_	437.0		1.4 (*M. smegmatis*)		42.6 (Vero cells)	30.43	
Suadamin A (**10**) (Alkaloid)	C_43_H_48_N_4_O_6_	716.88	*Melodinus suaveolens* (Hance) Champ, ex Benth	6.76 (*M. tuberculosis* H37Rv)	MABA	143.3 (VERO cells)	21.2	[58]
Suadamin B (**11**) (Alkaloid)	C_43_H_48_N_4_O_6_	716.88		33.47 (*M. tuberculosis* H37Rv)				
Decarine (**12**) (Alkaloid)	C_19_H_13_NO_4_	319.3	*Zanthoxylum capense* (Thunb.) Harv	5.01 (*M. tuberculosis* H37Rv)	Broth microdilution method	206.7 (THP-1)	41.2	[59]
				9.71 (*M. tuberculosis* H37Ra)			21.3	
(*2E*,*4E*)-N-isobutyl-2,4-tetradecadienamide (**13**) (Amide)	C_18_H_33_NO	279.46	*Z. capense* (Thunb.) Harv	5.73 (*M. tuberculosis* H37Rv)	Broth microdilution method	219 (THP-1)	38.2	[59]
Lassomycin (**14**) (Peptide)	C_82_H_142_N_30_O_20_	1868.23	*Lentzea kentuckyensis*	0.41–0.83 (*M. tuberculosis* H37Rv)	MABA	187.34 (Human NIH 3T3 and HepG2 cells)	225.72–456.93	[60]
				1.65 (*M. tuberculosis* H37Rv resistant to INH, RIF, STR, EMB, PZA, FQ)			113.5	
				0.07–0.13 *M. avium* subsp. *Paratuberculosis*			1441.1–2676	
				0.41–1.06 *M. smegmatis*			176.4–456.92	
Rufomycin I (**15**) (Peptide)	C_54_H_77_N_9_O_10_	1012.56	*Streptomyces* sp.	0.02 (*M. tuberculosis* H37Rv)	MABA	>50 (Vero cell)	>2500	[61]
				<0.004 (*M. tuberculosis* H37Rv resistant to INH				
				0.073 *M. smegmatis*				
				<0.02 (*M. bovis*)				
Ecumicin (**16**) (Peptide)	C_83_H_134_N_14_O_17_	1600.0	*Streptomyces* sp.	0.16 (*M. tuberculosis* H37Rv)	MABA	>63 (Vero cell line)	>3150	[61]
				<0.12 (*M. tuberculosis* H37Rv resistant to INH)				
				1.7 (*M. smegmatis*)				
				<0.2 *M. bovis*				
Cyclomarin A (**17**) (Peptide)	C_56_H_82_N_8_O_11_	1043.3	*Streptomyces* sp.	0.094 (*M. tuberculosis* H37Rv)	MABA	>50 (Vero cell line)	>531	[61]
				1.6 (*M. smegmatis*)				
				<0.02 (*M. bovis*)				
Caprazamycin B (**18**) (Peptide)	C_53_H_86_N_5_O_22_	1144.6	*Streptomyces* sp.	2.73 (*M. tuberculosis* H37Rv)		>4368.33 (Mice)	>1600	[62]
Eurylene (**19**) (Terpenoid)	C_34_H_58_O_8_	594.8	*Homalolepis suffruticosa* (Engl.) Devecchi & Pirani	2.35 (*M. tuberculosis* H37Rv)	MTT	67.41 (RAW 264.7)	28.7	[63]
				3.36 (*M. tuberculosis* H37Rv)		67.41 (RAW 264.7)	20.06	
12-Deacetoxyscalarin 19-acetate (**20**) (Terpenoid)	C_27_H_40_O_4_	428.6	*Brachiaster* sp.	4.00 (*M. tuberculosis* H37Ra)	MABA	Inactive in cytotoxicity assays against several cell lines MCF-7, HT-29, HeLa, KB		[64]
(−)-8,15-diisocyano-11(20)-amphilectene (**21**) (Terpenoid)	C_22_H_32_N_2_	324.51	*Svenzea flava*	9.8 (*M. tuberculosis* H37Rv)	Microbroth dilution assay	99.74 (Vero cells)	10.2	[65]
Saringosterol (**22**) (Steroid)	C_29_H_48_O_2_	428.73	*Lessonia nigrescens*	0.58 (*M. tuberculosis* H37Rv)	BACTEC 460	>298.55 (Vero cells)	>514.74	[66]
Stigmast-5-ene-3β-ol-7-one (**23**) (Steroid)	C_29_H_48_O_2_	428.73	*Thalia multiflora* Horkel ex Koernicke (*Marantaceae*)	4.62 (*M. tuberculosis* H37Rv)	MABA	>237.91 (Vero cells)	>51.5	[67]
Stigmast-4-ene-6β-ol-3-one (**24**) (Steroid)	C_29_H_46_O_2_	426.68		10.07 (*M. tuberculosis* H37Rv)		>239.05 (Vero cells)	>23.74	
Stigmast-5,22-diene-3β-ol-7-one (**25**) (Steroid)	C_29_H_46_O_2_	426.68		2.34 (*M. tuberculosis* H37Rv)		>239.05 (Vero cells)	>102.16	
Stigmast-4,22-diene-6β-ol-3-one (**26**) (Steroid)	C_29_H_46_O_2_	426.68		2.34 (*M. tuberculosis* H37Rv)		>239.05 (Vero cells)	>102.16	
11(S),16(R)-Dihydroxy-octadeca-9Z,17-dien-12,14-diyn-1-yl acetate (**27**) (Polyacetylene)	C_20_H_28_O_4_	332.40	*Angelica sinensis* (Oliv.) Diels	4.2 (*M. tuberculosis* Erdman)	MABA	>361.01		[68]
Falcarindiol (**28**) (Polyacetylene)	C_17_H_24_O_2_	260.40		23.4 (*M. tuberculosis* Erdman)		>460.83		
5,6-dehydro-7,8-dihydromethysticin (**29**) (Lactone)	C_15_H_14_O_5_	274.27	*Piper sanctum* (Miq.) Schltdl. ex C. DC	14.58 (*M. tuberculosis* H37Rv)	MABA	152.98 (Vero cells)	10.5	[69]
Micromolide (**30**) (Lactone)	C_18_H_32_O_2_	338.8	*Micromelum hirsutum* Oliv.	5.35 (*M. tuberculosis* H37Rv)	MABA	280.40 (Vero cells)	52.41	[70]
2-oxo-14-(3′,4′-methylenedioxyphenyl) tetradecane (**31**) (Aryl-alkyl ketone)	C_21_H_32_O_3_	332.23	*Piper sanctum* (Miq.) Schltdl. ex C. DC	18.81 (*M. tuberculosis* H37Rv)	MABA	>361.2 (Vero cells)	20.82	[69]
2-oxo-16-(3′,4′-methylenedioxyphenyl) hexadecane (**32**) (Aryl-alkyl ketone)	C_23_H_36_O_3_	360.26		17.35 (*M. tuberculosis* H37Rv)		>333.08 (Vero cells)	19.2	
Linoleic acid (**33**) (Fatty acid)	C_18_H_32_O_2_	280.4	*Warburgia ugandensis*	14.26 (*M. aurum*)	MTT	193 (Prostate cancer LNCaP cells)	13.53	[71]
				14.24 (*M. phlei*)		193 (Prostate cancer LNCaP cells)	13.53	
3-Nitropropionic (**34**) (Nitroacid)	C_3_H_5_NO_4_	119.08	*Phomopsis* sp. strain usia5	3.3 μM (*M. tuberculosis* H37Ra)	MABA	Inactive against Vero cell lines		[72]
Maritinone (**35**) (Naphthoquinone)	C_22_H_14_O_6_	374.34	*Diospyros anisandra* S.F.Blake	8.34 (*M. tuberculosis* H37Rv & MDR-MTB)	MABA	621.60 (Vero cells)	161.88	[73]
3,3’-biplumbagin (**36**) (Naphthoquinone)	C_22_H_14_O_6_	374.3		8.34 (*M. tuberculosis* H37Rv & MDR-MTB)		1623.22 (Vero cells)	194.63	

INH, isoniazid, MTT; 3-(4,5-dimethylthiazole-2-yl)-2,5-diphenyl tetrazolium bromide; MABA; Microplate Alamar Blue Assay.

## Data Availability

There is no additional data associated with this article.
